# Combined Effects of Different Alleles of *FLO*2, *Wx* and *SSIIa* on the Cooking and Eating Quality of Rice

**DOI:** 10.3390/plants11172249

**Published:** 2022-08-30

**Authors:** Yu Zhang, Jiajia Zhao, Yaqi Hu, Yanni Zhang, Yining Ying, Feifei Xu, Jinsong Bao

**Affiliations:** 1 Institute of Nuclear Agricultural Sciences, Key Laboratory for Nuclear Agricultural Sciences of Zhejiang Province and Ministry of Agriculture and Rural Affairs, Zhejiang University, Zijingang Campus, Hangzhou 310058, China; 2 Hainan Institute of Zhejiang University, Yazhou Bay Science and Technology City, Yazhou District, Sanya 572025, China

**Keywords:** floury endosperm, rice quality, *SSIIa*, starch, *Wx*

## Abstract

The improvement of the cooking and eating quality (CEQ) of rice is one of the major objectives of current rice-breeding programs. A few major genes such as *Waxy* (*Wx*) and *starch synthase IIa* (*SSIIa*) have been successfully applied in molecular breeding. However, their interactive effects on CEQ have not been fully understood. In this study, a recombinant inbred line (RIL) population was constructed by crossing the white-core mutant *GM*645 with the transparent phenotype of the japonica rice variety Tainung 67 (TN67). *GM*645 and TN67 contain different alleles of *FLOURY ENDOSPERM*2 (*FLO*2), *Wx*, and *SSIIa*. The effects of different allele combinations of *FLO*2, *Wx,* and *SSIIa* on the CEQ of rice were investigated. The inbred lines with the mutation allele *flo*2 had a significantly lower apparent amylose content (AAC), viscosity characteristics except for setback (SB), and gel texture properties compared to those lines with the *FLO*2 allele. The allelic combination of *FLO*2 and *Wx* significantly affected the AAC, breakdown (BD), and gel textural properties, which could explain most of the variations in those rice quality traits that were correlated with AAC. The allelic combination of *FLO*2 and *SSIIa* significantly affected the hot paste viscosity (HPV) and pasting temperature (PT). The *Wx* × *SSIIa* interaction had a significant effect on the PT. The interaction of *FLO*2, *Wx* and *SSIIa* significantly affected the AAC, cold paste viscosity (CPV), PT, and consistency viscosity (CS). These results highlight the important roles of these quality-related genes in regulating the CEQ of rice and provide new clues for rice-quality improvement by marker-assisted selection.

## 1. Introduction

Rice (*Oryza sativa* L.), with a long history of cultivation and consumption, can provide more than 20% of the required energy for the entire global population [1,2]. Given the improvement in rice production techniques and people’s quality of life as well as the change in consumption styles and habits, people have placed higher demands on rice quality [3,4]. Cooking and eating quality (CEQ) and appearance quality are highly valued by consumers [5]. CEQ is usually indirectly reflected by the apparent amylose content (AAC), the viscosity characteristics measured by a rapid viscosity analyzer (RVA), and textural parameters such as hardness and cohesiveness measured by a texture profile analyzer [6]. Appearance quality includes indicators such as chalkiness (percentage of chalky grains and the degree of chalkiness), grain length, grain width, and length to width ratio [7]. 

Starch is the most important compound component in rice seeds [8,9], which accounts for 75–85% of their dry weight and is related to the appearance, stickiness, hardness, palatability, and digestibility of cooked rice [10]. Starch consists of linear polysaccharides containing α-1,4 glycosidic bonds (amylose) and highly branched polysaccharides containing both α-1,4 and α-1,6 glycosidic bonds (amylopectin) [11,12]. Starch is synthesized in the endosperm by the action of ADP-glucose pyrophosphorylase (AGPase), starch synthases (SSs), branching enzymes (BEs), and debranching enzymes (DBEs). Among them, the granulated starch synthase (GBSS) encoded by the *Waxy* (*Wx*) gene is responsible for amylose synthesis, while SSs extend the glucan chains of starch linked by α-1,4 glycosidic bonds, BEs introduce the branching point of the α-1,6 glycosidic linkage, and finally starch relies on DBEs to remove incorrect branch points. The latter three kinds of enzymes work together to complete the synthesis of amylopectin [13,14,15]. 

In the rice germplasm, various *Wx* alleles have been identified, including *Wx^a^*, *Wx^b^*, *Wx^in^*, *Wx^op^*, *Wx^hp^*, *Wx^mq^*, *Wx^mp^*, *Wx^lv^,* and *wx* [16,17,18,19,20]. Among the non-glutinous rice varieties, *Wx^a^* and *Wx^b^* are the two main functional genes of *Wx* [2]. *Wx^a^*, which leads to a high AAC, is mainly found in *indica* rice; *Wx^b^*, which leads to a low or moderate AAC, is mainly found in *japonica* rice. Compared with *Wx^a^*, the single nucleotide polymorphism (SNP) G at the first intron splice site becomes T in *Wx^b^*. This mutation reduces the splicing efficiency of pre-mRNA and reduces the accumulation of GBSS, which in turn results in a lower AAC [21]. Multiple other alleles have been identified from different rice varieties in the last decades [18,19,20]. In addition to the above allelic variants, the 5′-untranslated region of *Wx* exon 1 also contains a (CT)n microsatellite locus, and the polymorphism of this locus correlates with the AAC [22,23,24]. The SS family, which is involved in the branching extension of amylopectin, contains the SSI, SSII, SSIII, and SSIV isoforms. Among them, the gene-encoding SSIIa, also known as the main effector gene in regulating gelatinization temperature [24,25], can extend the A and B1 short chains of DP <10 to form the B1 long chain of amylopectin [26]. The SNP variation of GC/TT in exon 8 has been found to have the greatest effect on pasting temperature (PT) [27,28], and varieties containing GC usually show a high PT, while varieties containing TT usually show a low PT [27].

The floury endosperm is very similar to the chalky phenotype in terms of the grains’ transparency and the starch granule morphology and arrangement, so it can be regarded as an extreme chalky phenotype [29]. Many floury mutant genes, such as *FLO*2, *FLO*4–8, *FLO*10–16, and *FLO*18–19, have been cloned and characterized [30,31,32,33,34,35,36,37,38,39,40,41,42,43,44,45]. Among them, the *FLO*2 is located on the chromosome 4 and encodes a protein with a tetratricopeptide repeat motif (TPR) domain containing three motifs, which can mediate protein interactions and is involved in regulating the expression of a variety of starch synthesis-related enzyme genes and storage protein genes [30]. The *FLO*2 mutant can reduce AAC and storage proteins, and alter the structure of amylopectin [30,46,47,48].

At present, a large volume of research has been conducted on the *Wx* and/or *SSIIa* genes regarding their functions and their allelic effects on grain quality [10,49,50], but no studies have been conducted that analyze the specific effects of different allele combinations of the *FLO*2, *Wx,* and *SSIIa* genes on the CEQ of rice. To this end, a recombinant inbred line (RIL) population of *GM*645/Tainung67 (TN67), obtained in a previous study [51], is employed to investigate the effects of different combinations of *Wx*, *SSIIa,* and *FLO*2 on the CEQ of rice. Our results will facilitate an understanding of the effects of gene interactions on rice starch synthesis and CEQ formation and provide new avenues for rice-quality improvement by marker-assisted selection.1

## 2. Results

### 2.1. Variation Analysis of Quality Traits in Parents and RIL

**Figure 1:**
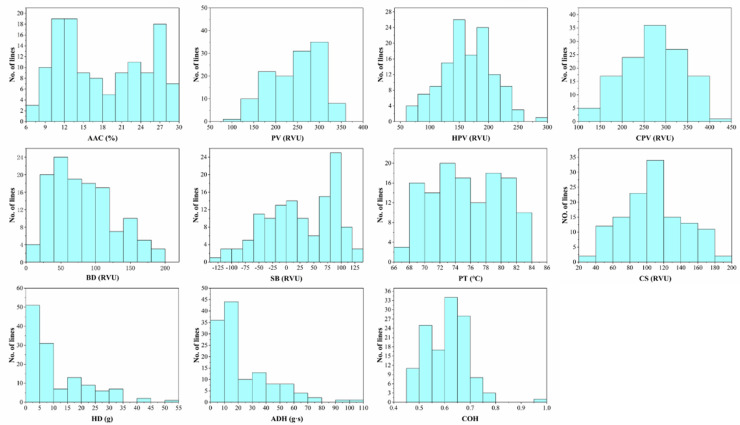
Distribution of apparent amylose content and RVA spectrum eigenvalues in RIL.

The eleven starch characteristics of the parents were analyzed, and significant differences were found between the two parents for seven traits: AAC, PV, SB, PT, HD, ADH, and COH (Table 1). The coefficient of variation of SB (247.8%) was the largest among all the traits, which ranged from −121.1 to 135.4 RUV. The smallest coefficient of variation was found in PT (6.1%), which ranged from 66.9 to 83.9 °C (Table 1). All the rice quality traits showed a continuous variation in the RIL population (Figure 1). A significant transgressive segregation was found for all the traits, and the mean values of the traits except the HPV, PT, CS, and HD were between the two parents (Table 1). The skewness and kurtosis for the PV, HPV, CPV, BD, SB, and CS were <1.0, which displayed an approximately normal distribution, indicating that these traits were quantitative traits that are controlled by multiple genes (Table 1; Figure 1). 

**Figure 2:**
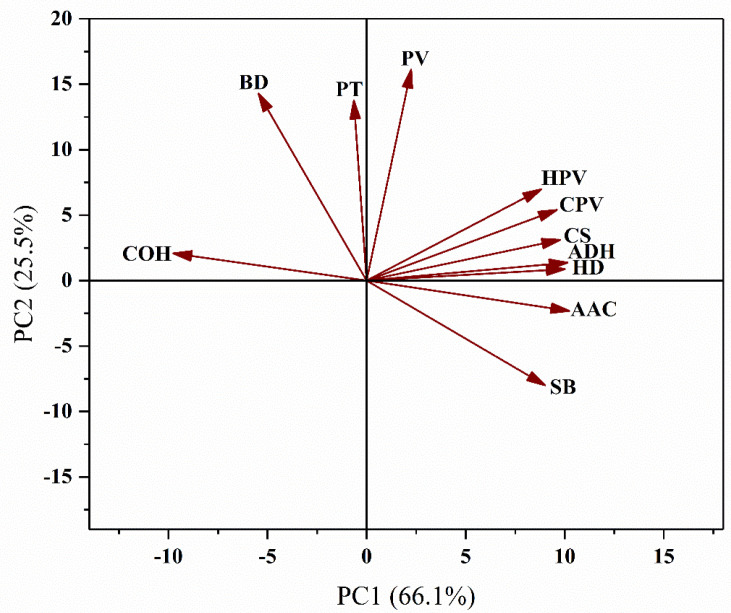
Principal component analysis of rice quality properties.

A principal component analysis was performed on the 11 rice quality traits (Figure 2). The PC1 and PC2 could explain 91.6% of the total variance. The AAC had the largest positive effect on PC1, and the HPV, CPV, CS, ADH, HD, and SB had a strong correlation with PC1. All the above seven traits were strongly correlated with each other. COH had a large negative effect on PC1, and negatively correlated with the seven traits above. The PV, BD, and PT had a large positive effect on PC2, and SB had the largest negative effect on PC2, so PV, BD, and PT were negatively correlated with SB.

### 2.2. Genotyping of RIL

**Figure 3:**
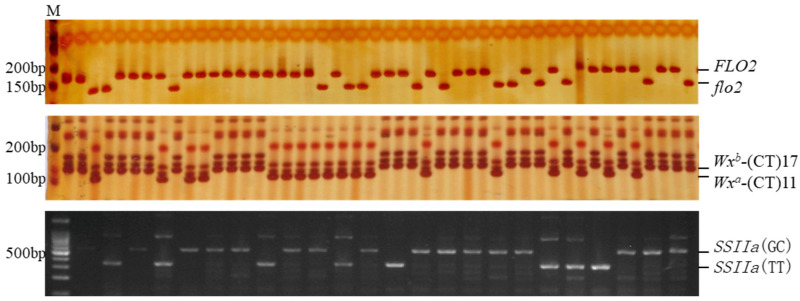
Genotyping of *FLO*2, *Wx,* and *SSIIa* alleles in RIL. M—DNA marker; bp—base pair.

The white-core phenotype of *GM*645 is caused by the presence of a 1 bp thymine deletion in exon 18 of the *FLO*2 gene, leading to an early appearance of the terminator codon [49]. The PCR product containing the thymine deletion can be specifically identified and cleaved by the *Hinf*I (Figure 3). Hereafter, *FLO*2 indicates the wild-type and *flo*2 indicates the mutant allele, so *GM*645 possesses the *flo*2 allele and TN67 possesses the *FLO*2 allele. Among the 127 RILs, 41 lines had the *flo*2 allele and 86 lines had the *FLO*2 allele. 

The white-core mutant *GM*645 had the *Wx^a^*-(CT)11 and *SSIIa(*GC) alleles, while the japonica Tainung67 (TN67) with the transparent endosperm had the *Wx^b^*-(CT)17 and *SSIIa(*TT) alleles. The polyacrylamide gel electrophoresis plots of the PCR products from the 127 RIL populations showed that there were 59 *Wx^a^* lines and 68 *Wx^b^* lines (Figure 3). The GC/TT polymorphism in exon 8 of *SSIIa* could be genotyped using four primers [28], and according to the results of agarose gel electrophoresis, there were 77 lines with the *SSIIa(*GC) and 50 lines containing the *SSIIa(*TT) in the RIL population (Figure 3).

### 2.3. Effects of FLO2, Wx, and SSIIa Single Gene on the AAC, Viscosity Properties, and Textural Properties in the RIL Population

*FLO*2 and its mutant allele *flo*2 had significant effects on nine traits (Table 2). Compared to *FLO*2, the RIL lines carrying the *flo*2 allele showed lower levels in all traits except COH (*p* < 0.05), indicating that the loss of function of *FLO*2 had a great impact on the starch-related physicochemical properties and the CEQ of rice.

*Wx* is the major gene regulating the AAC, viscosity properties, and textural properties in rice endosperm. *Wx^a^* maintained higher levels in most of the starch quality traits except for the CPV, PT, and COH. The different alleles of *SSIIa* only had a significant effect on HPV and PT (*p* < 0.05) but had no effects on the AAC and textural properties (Table 2).

### 2.4. Differences in the AAC, Viscosity and Textural Properties among Eight Genetic Combinations

Since *FLO*2, *Wx*, and *SSIIa* are genetically segregated in the RIL population, there may be specific interactive effects among these three genes. Theoretically, a total of eight genotype combinations can be generated. A total of 24 lines had the *FLO*2/*Wx^a^*/*SSIIa*(GC) combination (genotype), 18 had the *FLO*2*/Wx^a^/SSIIa(*TT) genotype, 24 had the *FLO*2/*Wx^b^*/*SSIIa*(GC) genotype, 20 the had *FLO*2/*Wx^b^*/*SSIIa*(TT) genotype, 13 had the *flo*2/*Wx^a^*/*SSIIa*(GC) genotype, 4 had the *flo*2/*Wx^a^*/*SSIIa*(TT) genotype, 16 had the *flo*2/*Wx^b^*/*SSIIa*(GC) genotype, and 8 had the *flo*2/*Wx^b^*/*SSIIa*(TT) genotype. The starch quality traits among the eight combinations are shown in Table 3.

Among the eight combinations, all eleven starch quality traits were different (*p* < 0.05), indicating that there were significant interactions between *FLO*2*, Wx,* and *SSIIa*. In terms of the AAC, the *FLO*2*/Wx^a^/SSIIa(*TT) genotype had the highest average value (26.23 ± 2.67%), and the lowest average value was in the *flo*2*/Wx^b^/SSIIa(*TT) genotype (10.87 ± 2.08%). The *SSIIa(*TT) allele was involved in both combinations, suggesting that *SSIIa* may have a weak effect on the AAC. In terms of viscosity characteristics, the BD (136.0 ± 35.1 RVU) and PT (81.7 ± 1.2 °C) were highest in the *FLO*2*/Wx^b^/SSIIa(*GC) genotype. The PVs of the *FLO*2*/Wx^b^/SSIIa*(TT) genotype (289.7 ± 35.4 RVU) and *FLO*2*/Wx^b^/SSIIa*(GC) genotype (283.3 ± 48.3 RVU) were the highest. The highest CPV (345.4 ± 25.1 RVU) and CS (152.1 ± 19.6 RVU) were found in the *FLO*2*/Wx^a^/SSIIa*(GC) genotype, but the *FLO*2*/Wx^a^/SSIIa*(TT) genotype had the same CPV (343.5 ± 40.6 RVU). The *FLO*2*/Wx^a^/SSIIa(*TT) combination showed the highest HPV (214.5 ± 38.7 RVU). The *flo*2*/Wxa/SSIIa*(TT) combination had the highest SB (97.4 ± 6.0 RVU). Most of the lowest average values appeared in the *flo*2*/Wx^b^/SSIIa*(TT) combination, except for the BD and SB. In terms of textural properties, the highest values of the two traits, HD and ADH, appeared in the *FLO*2*/Wx^a^/SSIIa*(GC) combination, while the lowest average values appeared in the *flo*2*/Wx^b^/SSIIa*(TT) combination. The highest average value of COH belongs to the *FLO*2*/Wx^b^/SSIIa*(TT) combination.

### 2.5. Effects of Interaction between FLO2, Wx, and SSIIa on AAC, Viscosity Properties, and Textural Properties of RIL

**Figure 4:**
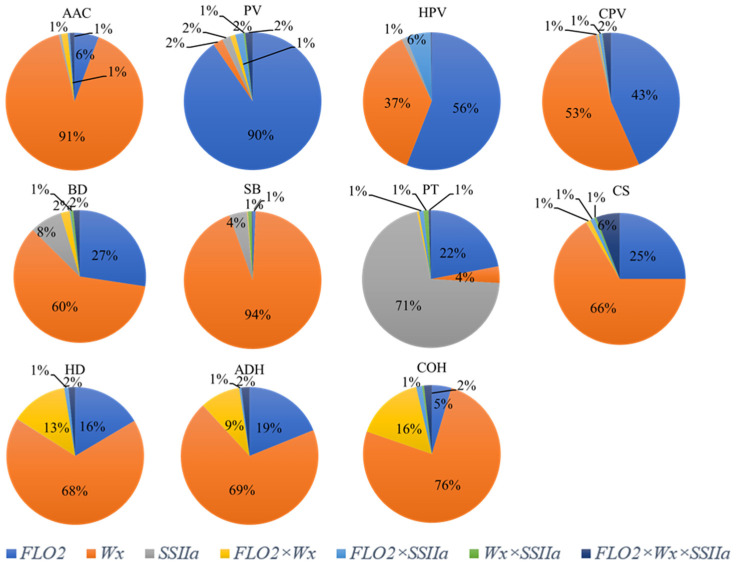
Percentages of variance of starch properties in RIL population explained by the *FLO*2, *Wx* and *SSIIa* interactions.

The analysis of the interactions between the genes for the variation in the starch properties was carried out by ANOVA, and the results are shown in Figure 4. The *Wx* gene accounted for more than 91% of the total variation of AAC, so the AAC was mainly controlled by the *Wx* gene. Similarly, *Wx* could explain more than 53% of the variation in the CPV, BD, SB, CS, HD, ADH, and COH, indicating that those traits were also controlled by the *Wx* and had a close correlation with the AAC. *FLO*2 accounted for more than 90% of the PV variation and was the only factor that had a significant effect on the PV (*p* < 0.001). *FLO*2 also explained 56% of the total variation in the HPV, 43% of the total variation in the CPV, and 16–27% of the total variation in the BD, PT, CS, HD and ADH, indicating that the viscosity and gel textural traits were also controlled by *FLO*2. *SSIIa* was responsible for PT, which explained 71% of the total variation.

Significant interactions between the genes were detected. Among the interactions, the *FLO*2 × *Wx* interaction accounted for 9–16% of the total variation in the gel textural properties. The *FLO*2 × *Wx* interaction was also significant for AAC and BD, but it only explained less than 2% of the total variation.

*FLO*2 × *SSIIa* interaction accounted for 6% of the total variation in the HPV. Although the interaction was significant for the PT, it only explained around 1% of the total variation. The *Wx* × *SSIIa* interaction was only detected in the PT, explaining less than 1% of the total variation. A significant *FLO*2 × *Wx* × *SSIIa* interaction was detected for most traits (Figure 4). This triple interaction could explain 6% of the total variation in the CS, and around 2% of the total variation in the PV, CPV, BD, HD, ADH, and COH, and less than 1% of the total variation of other traits. 

### 2.6. Cluster Analysis Based on the Quality Traits of RIL Population

**Figure 5:**
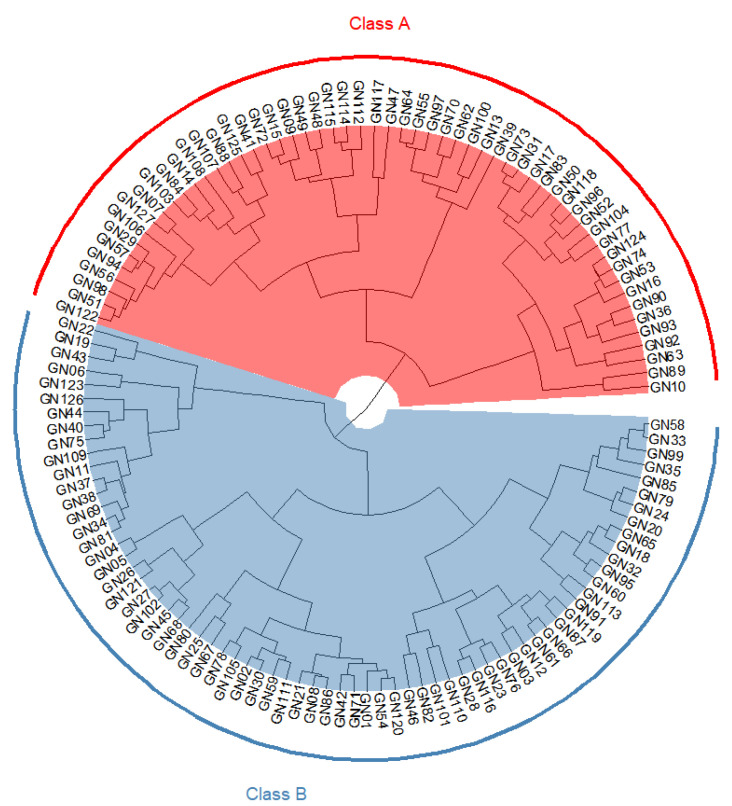
Cluster analysis of 11 quality traits in RIL population. The red part represents Class A, including 59 RIL lines involving four allele combinations of *FLO*2*/Wxa/SSIIa(*GC), *FLO*2*/Wxa/SSIIa(*TT), *flo*2*/Wxa/SSIIa(*GC), and *flo*2*/Wxa/SSIIa(*TT). The blue part represents Class B, including 68 RIL lines involving four allele combinations of *FLO*2*/Wxb/SSIIa(*GC), *FLO*2*/Wxb/SSIIa(*TT), *FLO*2*/Wxb/SSIIa(*GC), and *FLO*2*/Wxb/SSIIa(*TT).

A cluster analysis was performed using hierarchical clustering with all 11 starch quality traits as variable indicators. The 127 populations of RIL were divided into two classes (Figure 5). When analyzed in combination with the genotype combinations contained in the 127 populations, Class A was found to contain *FLO*2/*Wx^a^*/*SSIIa(*GC), *FLO*2/*Wx^a^*/*SSIIa(*TT), *flo*2/*Wx^a^*/*SSIIa(*GC), and *flo*2/*Wx^a^*/*SSIIa(*TT); Class B contained *FLO*2/*Wx^b^*/*SSIIa(*GC), *FLO*2/*Wx^b^*/*SSIIa(*TT), *flo*2/*Wx^b^*/*SSIIa(*GC), and *flo*2/*Wx^b^*/*SSIIa(*TT). The clustering results showed that the classification of the RIL population basically depended on the genotype of *Wx*. Class A contained lines with a high AAC, while class B contained lines with a low to medium AAC. This also proved that *Wx* was the most important gene regulating the CEQ traits of rice.

## 3. Discussion

### 3.1. FLO2 Affects Rice Quality

The quality of rice, especially the CEQ, plays a crucial role in the market value of rice. It has been found that a series of genes directly or indirectly involved in starch synthesis can regulate the quality of rice [52,53]. Among them, *FLO*2 plays a key role in the regulation of rice grain size and starch quality by regulating the expression levels of starch synthesis-related genes and storage protein genes in the endosperm [30,46,47]. *FLO*2 contains a total of 23 exons and 22 introns, encoding a protein with two domains: one is a TPR domain containing three repeats of the TPR motif, and the other is an unproven protein clueless (CLU) domain containing 245 amino acid residues [30,54]. The TPR domain is a mediator domain that is completely composed of a helical structure and participates in the interaction between proteins. It plays an important role in the formation of protein complexes. Different motifs in the same protein have different effects and different characteristics [55,56]. The CLU domain is a large and highly conserved protein involved in normal mitochondrial function [57].

This study found that the AAC decreased in the RIL population lines containing the *flo*2 mutant allele, which is consistent with previous findings [30,48], indicating that *FLO*2 had an effect on amylose synthesis. Compared with the wild-type Guangluai4 (GLA4), the *GM*645 carrying the *flo*2 mutant allele only reduced the AAC by 6%, and the difference between the two accessions did not reach a significant level [48]. In this study, under the same *Wx* background, the *flo*2 allele can reduce the AAC by 17% in the *Wx^a^* background and 11% in the *Wx^b^* background (Table 3). However, in previous studies, other *flo*2 alleles showed a very significant effect on the AAC. She et al. [30] identified a *flo*2 allele with a mutated site in exon 14 and found the AAC decreased by about 40% compared to the wild-type. Wu, et al. [46] reported three *flo*2 alleles with mutations in exon 19, intron 9, and intron 11, and found that the AAC decreased by 24%−26% compared to the wild-type. Qiu [54] identified nine *flo*2 alleles with mutation sites in exons 6, 10, 11, and 21 and introns 13, 14, 16, and 17. The AAC was reduced by about 67%−73%. These evidences imply that different mutation sites in *FLO*2 will lead to different degrees of influence on the AAC. This may be due to the different effects of mutations on the TPR domain and the CLU protein domain, and different TPR motifs may have different roles in regulating amylose synthesis. The phenotypic differences caused by different allelic mutants have also been reported in *flo4* and *flo6* [58].

All the viscosity characteristics of the RIL population containing the *flo*2 allele were significantly lower than those of the line containing the *FLO*2 allele except SB, which was consistent with the results of previous studies [46,48]. The viscosity characteristics of rice are usually correlated with the AAC. Our principal component analysis also confirmed that the AAC was significantly correlated with most RVA viscosities except the BD and PT (Figure 2). Thus, the effect of *FLO*2 on the RVA viscosity was mainly due to its effect on the AAC. However, the fine structure of amylopectin also has an important impact on the RVA profile. Studies have shown that the short chain in amylopectin (DP 6–12) reduces the SB and CPV by reducing the rate of molecular polymerization [59]. The longer the branched chains are, the more easily they are intertwined with each other, which is conducive to maintaining the integrity of starch granules and reducing the capacity for water absorption and swelling, resulting in a lower BD, PV, and HPV [60,61]. Compared with the *FLO*2 from GLA4, the *flo*2 from *GM*645 increased the short chain (DP 6–9 and DP 22–35) and ultra-long chain DP ≥ 44 [48]. Combined with the change in the RVA profile, it is the *flo*2 mutation that caused the change in the amylopectin side chain distribution and then the change of RVA viscosity parameters. However, She et al. [30] showed that a DP ≤ 9 and DP 22–28 in the amylopectin short chain of the mutant increased, while the DP 9–21 and long chain (DP ≥ 38) decreased, suggesting that different *FLO*2 mutation sites may have different effects on the fine structure of amylopectin. In *flo*2 mutants, the expression of genes related to starch synthesis, such as *AGPS*1, *GBSSI*, *SSIIa*, *BEI*, *BEIIa*, *BEIIb*, *PUL,* and *ISA*1, were down regulated [30,62], which can explain why the AAC decreased and why the amylopectin chain length distribution changed. This study also found that *FLO*2 has the most significant effect on the PV (Figure 4), explaining more than 90% of the total variation (Figure 4). The PV indicates the swelling degree of starch particles and their ability to bind to water, which is related to the AAC, amylopectin fine structure, and non-carbohydrate components (lipids and proteins) in starches. Therefore, *FLO*2 plays a unique role in the PV.

The texture of rice gel is mainly affected by the content and morphology of starch, protein, non-starch polysaccharides, and other substances [63]. Most of the texture parameters in the *FLO*2 mutant were lower than those in the wild-type. In addition to the reduced AAC invoked in the *flo*2 mutant, other studies have confirmed that the total starch content and total protein content were decreased to some degrees, but that the soluble sugar content was increased [54]. The AAC is negatively correlated with COH, and positively correlated with HD and ADH (Figure 2). According to the results of this study, the effect of *FLO*2 on rice gel texture was due to its effect on the AAC. Whether *FLO*2 could regulate the texture characteristics by affecting the amylopectin fine structure or protein content needs to be further explored.

### 3.2. Interaction Effect of FLO2, Wx and SSIIa

The rice CEQ is regulated by a series of genes. The interactive effects between different genes/enzymes are diverse and complex, forming a complex regulatory network [15,64,65,66]. As a coding gene of GBSSI, *Wx* can regulate the content of amylose in rice, which is related to a variety of rice-quality indicators and plays an important role in the CEQ of rice. Among the 11 CEQ parameters, *Wx* had a significant effect on 10 traits except for PV (Figure 4). The cluster analysis revealed that the grouping of RIL lines was based on the different alleles of *Wx* (Figure 5). In addition to *Wx*, CEQ is also regulated by other starch synthesis-related genes such as *SSIIa*. However, this study found that *SSIIa* only had a significant effect on the AAC, BD, SB, and PT.

The *FLO*2 × *Wx* gene interaction has a significant impact on the AAC, BD, HD, ADH and COH. This gene combination can explain most of the changes in rice quality traits involving the AAC. The *FLO*2 × *SSIIa* gene interaction only has a significant effect on the HPV and PT. *Wx* is the main gene regulating the AC and gel consistency, and it is also the minor gene affecting the gelatinization temperature (GT) [49]. On the contrary, *SSIIa* mainly regulates the GT, but also has a certain effect on the AC and GC [49]. In previous studies, *Wx* × *SSIIa* was considered to have a great impact on RVA characteristics [10]. Our study found that the interaction between *Wx* and *SSIIa* only has a significant effect on the PT.

*FLO*2 and *flo*2 have different effects on rice quality traits under different allele backgrounds of *Wx* and *SSIIa.* Under the condition of the same *SSIIa* allele, in the *Wx^a^* background, a total of 10 traits, including the AAC, PV, HPV, BD, CPV, PT, CS, HD, ADH, and COH, were affected differently by *FLO*2 and *flo*2 (Table 3). In the background of *Wx^b^*, the PV, HPV, BD, CPV, PT, and CS were significantly affected (Table 3). With the same *Wx* allele, when in the background of *SSIIa(*TT), the RILs containing *FLO*2 had a higher AAC, PV, HPV, BD, CPV, PT, CS, and ADH than those RILs containing *flo*2 (Table 3). In the background of *SSIIa(*GC), the PV, HPV, BD, CPV, PT, CS, HD, and ADH were significantly different between *FLO*2 and *flo*2 (Table 3). These results indicated that there are significant interactions between *FLO*2, *Wx* and *SSIIa*. As expected, the ANOVA revealed that the *FLO*2 × *Wx* × *SSIIa* effects reached significant levels for the AAC, CPV, PT, and CS.

High quality rice should generally have a good palatability with a soft and elastic texture. Cooked rice with a 14%–20% medium amylose content will have a fluffy and soft texture. This study found that among the eight allele combinations composed of three genes, the *FLO*2/*Wx^b^*/*SSIIa(*TT) has the lowest AAC, PT, and HD, implying that it has a soft texture and poor retrogradation. This allele combination is expected to have the best CEQ.

In this study, it was found that single gene *FLO*2, *Wx,* and *SSIIa* are the main factors affecting the variation in all the rice quality traits. The *FLO*2 and *Wx* genes have significant effects on the variation of most viscosity and texture properties, which is mainly due to the fact that amylose content is mainly regulated by both *FLO*2 and *Wx*. However, the interactive effects are also significant for many CEQ traits. The *FLO*2 × *Wx* allele combination showed much greater effects on quality traits than the *FLO*2 × *SSIIa* and *Wx* × *SSIIa*. It is important to explore the allelic combinations of these genes for the improvement of the CEQ of rice.

## 4. Materials and Methods

### 4.1. Plant Materials

The white-core endosperm mutant *GM*645 was obtained from induced mutation breeding of the indica rice (*Oryza sativa* L.) variety Guangluai4 (GLA4) [48]. The white-core endosperm phenotype is caused by the deletion of a T nucleotide in exon 18 of the *FLO*2 gene [51]. A recombinant inbred line (RIL) population with 127 lines was constructed from the cross between *GM*645 and japonica Tainung67 (TN67) with transparent endosperm. The F_7_ lines were harvested in Sanya, Hainan Province, in April 2021.

### 4.2. Preparation of Rice Flour

The paddy was dried in the sun until the moisture content was about 12%, and then stored at room temperature for three months. The rice grains were dehulled (Type THU, Satake Co., Tokyo, Japan), polished (Type TM05C, Satake Manufacturing, Suzhou, China), and then ground to flour (Cyclone Sample Mill, UDY Corporation, Fort Collins, CO, USA) to pass through a 100-mesh sieve.

### 4.3. DNA Extraction and Genotyping

Five rice seeds of each line and parents were germinated. Genomic DNA was extracted from the leaves of 10-day-old seedlings using the CTAB method. The deletion of 1 bp thymine on exon 18 of the *FLO*2 gene was detected using the method described in [51] with specific primers (5’TGAACCAGCGTAACGACATTGTG3’ and 5’TATGAAGAGAGTTACGGGGATTTATCTGACT3’). The PCR products were digested with restriction endonuclease *Hinf*I for 1 h and separated on an 8% polyacrylamide gel for around 3h. The primers used to amplify the (CT)n microsatellite in the *Wx* gene were as follows: 5′CTTTGTCTCTCTCTCTCAGACAC3′ (484) and 5′TTGCAGATTTCTCTTCGATG3′ (485) [23,24]. The PCR products were also separated on the 8% polyacrylamide gels. For genotyping of the *SSIIa* allele, four of the allele-specific primers were used in a simple PCR (CGAGCGCACACACAG, GGCCGTGCAGATTAACCAT, CAAGAGAGGCTGGAGGGGC, and ACATGCGCACCTGGAAA) [28]. The PCR products were separated on a 2% agarose gel for around 2 h.

### 4.4. Apparent Amylose Content (AAC)

AAC was measured using a method described in Bao et al. [50]. The absorbance of the solution was measured at 620 nm using a Microplate spectrophotometer (Epoch, Biotek, Winooski, VT, USA). AAC was calculated using a standard curve made from five rice samples with known AAC.

### 4.5. Pasting Viscosity

The Pasting viscosity of rice flour was determined using Rapid visco analyser (RVA, Model 4500, Perten Instrument, Hägersten, Sweden). Three grams of rice flour (12% m.b.) were mixed with twenty-five grams of ddH_2_O in an aluminum RVA sample can. The heating and cooling cycle program was set as follows. The initial temperature was 50 °C. After 60 s, the sample was heated to 95 °C at a rate of 12 °C/60 s, maintained for 150 s, and then cooled to 50 °C with the cooling rate the same as the heating rate. The total program time was 12 min and 30 s. The starting speed of the RVA paddle was 960 rpm, which was reduced to 160 rpm after 15 s and maintained until the end of the program. The peak viscosity (PV), hot paste viscosity (HPV), cool paste viscosity (CPV), breakdown (BD, = PV – HPV), setback (SB, = CPV – PV), consistency (CS, = CPV – HPV) and pasting temperature (PT) were directly read out or calculated by TCW3 (Thermoline for Windows) software.

### 4.6. Gel textural Properties

The aluminum cans with rice flour gels were sealed by Parafilm after the RVA analysis and stored at 4 °C for 24 h. Texture characteristics were measured by a texture analyzer (TA.XTC-18, Shanghai Bosin Industrial Development Co., Shanghai, China) using a standard two cycle TPA program. A 5 mm diameter probe was used to compress the gel for 10 mm at 1 mm/s test speed. The hardness (HD, g), adhesiveness (ADH, g·s), and cohesiveness (COH) were derived from the software of the instrument.

### 4.7. Data Analysis

All the measurements were carried out at least in duplicate. Results were expressed as mean ± standard deviation (SD), and data were processed using analysis of variance (ANOVA) and multiple comparisons (Duncan’s multiple range test method) to determine significant differences by SAS (Version 9.3). The distribution of the data was determined, and principal component analysis was carried out using Origin 2017. The cluster analysis was carried out using the hclust function of the ggtree software package of R4.1.1 statistical software. The pie charts were created in Office 2019.

## Figures and Tables

**Table 1:** Rice-quality properties of parents and RIL.

Parameter	Parents		RIL				
	GM645	TN67	Mean ± SD	CV (%)	Range	Skewness	Kurtosis
AAC (%)	22.6 ^A^	16.3 ^B^	18 ± 6.8	37.8	6.7–29.1	0.2	−1.5
PV (RUV)	215.3 ^B^	269.9 ^A^	246.5 ± 56.4	22.9	96.5–351.3	−0.4	−0.7
HPV (RUV)	176.7 ^A^	171.1 ^A^	163.7 ± 43.1	26.3	62.3–281.7	0.1	−0.3
CPV (RUV)	279.1 ^A^	262.9 ^A^	271.7 ± 69.8	25.7	115.4–412.7	−0.3	−0.6
BD (RUV)	38.6 ^A^	98.8 ^A^	82.8 ± 44.0	53.1	11.3–198.2	−0.3	−0.5
SB (RUV)	63.8 ^A^	−7.0 ^B^	25.2 ± 62.5	247.8	−121.1 to 135.4	−0.3	−0.9
PT (°C)	72.8 ^B^	73.6 ^A^	75.5 ± 4.6	6.1	66.9–83.9	0.0	−1.2
CS (RUV)	102.4 ^A^	91.8 ^A^	108.0 ± 34.9	32.4	34.7–182.6	0.1	−0.6
HD (g)	11.5 ^A^	7.5 ^B^	11.6 ± 10.4	89.7	1.37–52.72	1.5	1.9
ADH (g·s)	24.8 ^A^	10.7 ^B^	23.2 ± 20.6	88.8	0.00–103.4	1.5	1.9
COH	0.58 ^B^	0.76 ^A^	0.61 ± 0.08	13.1	0.45–0.96	0.5	1.7

Different letters after the values between parents represent significant differences (*p* < 0.05). SD—standard deviation; CV—coefficient of variation.

**Table 2:** Effects of different alleles of *FLO*2, *Wx* and *SSIIa* on rice quality properties.

Parameter	*FLO*2		*Wx*		*SSIIa*	
	*FLO*2 (86)	*flo*2 (41)	*Wx^a^* (59)	*Wx^b^* (68)	TT (50)	GC (77)
AAC (%)	19.2 ± 7.1 ^A^	15.6 ± 5.2 ^B^	24.6 ± 3.1 ^A^	12.3 ± 2.6^B^	18.7 ± 6.7 ^A^	17.6 ± 6.7 ^A^
PV (RUV)	274.2 ± 40.4 ^A^	188.4 ± 37.8 ^B^	239.0 ± 46.7 ^A^	253.8 ± 62.9 ^A^	248.8 ± 60.9 ^A^	245.1 ± 53.2 ^A^
HPV (RUV)	180.6 ± 37.7 ^A^	128.4 ± 30.4 ^B^	188.1 ± 37.5 ^A^	142.6 ± 35.9 ^B^	175.4 ± 50.5 ^A^	156.2 ± 35.6 ^B^
CPV (RUV)	93.6 ± 43.8 ^A^	60.0 ± 34.7 ^B^	50.9 ± 22.9 ^B^	110.5 ± 38.9 ^A^	73.4 ± 40.7 ^A^	88.9 ± 45.0 ^A^
BD (RUV)	300.2 ± 57.3 ^A^	212.0 ± 54.2 ^B^	321.8 ± 46.8 ^A^	228.3 ± 56.1 ^B^	284.0 ± 71.2 ^A^	263.8 ± 67.8 ^A^
SB (RUV)	26.0 ± 64.8 ^A^	23.6 ± 57.3 ^A^	82.8 ± 21.1 ^A^	−24.8 ± 39.1 ^B^	35.2 ± 54.1 ^A^	18.7 ± 66.6 ^A^
PT (°C)	76.6 ± 4.7 ^A^	73.3 ± 3.7 ^B^	74.6 ± 4.1 ^B^	76.3 ± 4.9 ^A^	71.0 ± 2.3 ^B^	78.4 ± 3.2 ^A^
CS (RUV)	119.6 ± 31.2 ^A^	83.7 ± 29.4 ^B^	133.7 ± 27.6 ^A^	85.7 ± 23.5 ^B^	108.6 ± 30.9 ^A^	107.6 ± 37.3 ^A^
HD (g)	13.9 ± 11.5 ^A^	6.8 ± 4.2 ^B^	19.7 ± 10.3 ^A^	4.5 ± 1.0 ^B^	11.1 ± 9.3 ^A^	11.9 ± 10.9 ^A^
ADH (g·s)	27.9 ± 22.5 ^A^	13.3 ± 10.3 ^B^	38.8 ± 20.9 ^A^	9.7 ± 4.2 ^B^	22.7 ± 18.5 ^A^	23.5 ± 21.8 ^A^
COH	0.60 ± 0.08 ^A^	0.63 ± 0.08 ^A^	0.55 ± 0.06 ^B^	0.65 ± 0.06 ^A^	0.61 ± 0.07 ^A^	0.60 ± 0.08 ^A^

The number after the genotype indicates the number of lines with this allele in the RIL population. Different letters after the values between two alleles of the same gene represent significant differences (*p* < 0.05).

**Table 3:** Differences in rice quality traits in eight combinations of *FLO*2, *Wx,* and *SSIIa*.

Genetic Combination	AAC (%)	PV (RUV)	HPV (RUV)	BD (RUV)	CPV (RUV)	SB (RUV)
*FLO*2/*Wx^a^*/*SSIIa(*GC)	25.70 ± 2.07 ^A^	264.5 ± 28.6 ^A^	193.3 ± 23.5 ^AB^	71.2 ± 17.8 ^D^	345.4 ± 25.1 ^A^	80.9 ± 22.7 ^A^
*FLO*2/*Wx^a^*/*SSIIa(*TT)	26.23 ± 2.67 ^A^	257.9 ± 38.0 ^A^	214.5 ± 38.7 ^A^	43.4 ± 13.1 ^EF^	343.5 ± 40.6 ^A^	85.6 ± 24.4 ^A^
*FLO*2/*Wx^b^*/*SSIIa(*GC)	11.30 ± 1.94 ^D^	283.3 ± 48.3 ^A^	147.3 ± 21.7 ^D^	136.0 ± 35.1 ^A^	239.0 ± 33.8 ^C^	−44.4 ± 39.7 ^D^
*FLO*2/*Wx^b^*/*SSIIa(*TT)	14.41 ± 2.45 ^C^	289.7 ± 35.4 ^A^	174.8 ± 30.7 ^BC^	114.9 ± 27.6 ^B^	280.5 ± 38.7 ^B^	−9.2 ± 31.9 ^BC^
*flo*2/*Wx^a^*/*SSIIa(*GC)	20.89 ± 1.62 ^B^	184.8 ± 22.2 ^BC^	154.2 ± 24.0 ^CD^	30.6 ± 10.5 ^F^	262.8 ± 24.1 ^BC^	78.1 ± 11.1 ^A^
*flo*2/*Wx^a^*/*SSIIa(*TT)	22.20 ± 1.59 ^B^	176.8 ± 13.0 ^BC^	148.1 ± 10.4 ^D^	28.6 ± 11.4 ^F^	274.1 ± 14.2 ^B^	97.4 ± 6.0 ^A^
*flo*2/*Wx^b^*/*SSIIa(*GC)	12.01 ± 2.39 ^D^	207.5 ± 37.0 ^B^	115.5 ± 15.9 ^E^	92.0 ± 27.4 ^C^	179.4 ± 30.3 ^D^	−28.1 ± 36.0 ^BC^
*flo*2/*Wx^b^*/*SSIIa(*TT)	10.87 ± 2.08 ^D^	161.9 ± 46.8 ^C^	102.2 ± 30.8 ^E^	59.7 ± 21.8 ^DE^	163.7 ± 40.1 ^D^	1.7 ± 25.3 ^B^
Genetic Combination	PT (°C)	CS (RUV)	HD (g)	ADH (g·s)	COH	
*FLO*2/*Wx^a^*/*SSIIa*(GC)	79.0 ± 1.4 ^B^	152.1 ± 19.6 ^A^	25.14 ± 10.38 ^A^	48.68 ± 21.91 ^A^	0.52 ± 0.04 ^E^	
*FLO*2/*Wx^a^*/*SSIIa*(TT)	70.7 ± 1.8 ^E^	129.0 ± 29.0 ^B^	21.21 ± 7.78 ^A^	41.95 ± 16.23 ^A^	0.55 ± 0.05 ^DE^	
*FLO*2/*Wx^b^*/*SSIIa(*GC)	81.7 ± 1.2 ^A^	91.7 ± 16.0 ^D^	4.70 ± 0.91 ^CD^	10.25 ± 2.58 ^CD^	0.66 ± 0.04 ^AB^	
*FLO*2/*Wx^b^*/*SSIIa(*TT)	72.8 ± 1.6 ^D^	105.7 ± 13.6 ^CD^	4.72 ± 0.76 ^CD^	11.59 ± 3.60 ^CD^	0.67 ± 0.04 ^A^	
*flo*2/*Wx^a^*/*SSIIa(*GC)	73.8 ± 1.2 ^D^	108.7 ± 14.3 ^C^	9.64 ± 3.45 ^BC^	19.96 ± 9.77 ^BC^	0.61 ± 0.07 ^BC^	
*flo*2/*Wx^a^*/*SSIIa(*TT)	68.7 ± 0.9 ^F^	126.0 ± 15.5 ^B^	12.70 ± 5.46 ^B^	25.87 ± 10.99 ^B^	0.58 ± 0.05^CD^	
*flo*2/*Wx^b^*/*SSIIa(*GC)	76.5 ± 2.0 ^C^	63.9 ± 18.0 ^E^	4.48 ± 1.34 ^CD^	8.49 ± 5.66 ^D^	0.64 ± 0.10^AB^	
*flo*2/*Wx^b^*/*SSIIa(*TT)	68.2 ± 1.0 ^F^	61.4 ± 12.3 ^E^	3.71 ± 0.52 ^D^	5.65 ± 2.36 ^D^	0.65 ± 0.06^AB^	

Different letters after the values in each column indicated significant differences (*p* < 0.05).

## Data Availability

Data are contained within the article.

## Abbreviations

AAC	Apparent amylose content
ADH	Gel adhesiveness
BD	Breakdown
CPV	Cold paste viscosity
COH	Gel cohesiveness
CS	Consistency viscosity
CEQ	Cooking and eating quality
FLO	Floury
HD	Gel hardness
GT	Gelatinization temperature
HPV	Hot paste viscosity
PC	Principal component
PCA	Principal component analysis
PT	Pasting temperature
PV	Peak viscosity
RIL	Recombinant inbred line
RVA	Rapid Visco-Analyzer
RVU	Rapid Visco Unit
SB	Setback
SNP	Single nucleotide polymorphism
SS	Soluble starch synthase
Wx	Waxy
